# The prognostic value of CXC-chemokine receptor 2 (CXCR2) in gastric cancer patients

**DOI:** 10.1186/s12885-015-1793-9

**Published:** 2015-10-23

**Authors:** Zhenglin Wang, Hao Liu, Zhenbin Shen, Xuefei Wang, Heng Zhang, Jing Qin, Jiejie Xu, Yihong Sun, Xinyu Qin

**Affiliations:** 1 Department of General Surgery, Zhongshan Hospital, Fudan University, 180 Feng Lin Road, Shanghai, 200032 China; 2 Department of Gastroenterological Surgery, The First Affiliated Hospital of Dalian Medical University, Dalian, 116011 China; 3 Department of Biochemistry and Molecular Biology, School of Basic Medical Sciences, Fudan University, PO Box 103, 138 Yi Xue Yuan Road, Shanghai, 200032 China

**Keywords:** Gastric cancer, CXC chemokine receptor 2, Prognosis, Nomogram, Overall survival

## Abstract

**Background:**

CXC chemokine receptor 2 (CXCR2) has been reported to play an important role in the proliferation and invasion of gastric cancer cells. The present study aims to investigate the impact of CXCR2 expression on the overall survival (OS) of gastric cancer patients after radical resection.

**Methods:**

Intratumoral CXCR2 expression was evaluated with immunohistochemistry on tissue microarrays containing tumor samples of 357 gastric cancer patients from a single center. CXCR2 expression levels were correlated to clinicopathological variables and OS.

**Results:**

CXCR2 expression was mainly located in the cytoplasm of gastric carcinoma cells. High CXCR2 expression was associated with poor tumor differentiation (*p* = 0.021), increased tumor depth (*p* < 0.001), lymph node metastasis (*p* < 0.001), advanced TNM stage (*p* < 0.001) and short OS (*p* = 0.001). CXCR2 expression was an independent prognostic factor for OS (*p* = 0.001) in multivariate analysis, and could be combined with TNM stage to generate a predictive nomogram for clinical outcome in patients with gastric cancer.

**Conclusion:**

Intratumoral CXCR2 expression is a novel independent predictor for survival in gastric cancer patients. CXCR2 might be a promising therapeutic target of postoperative adjuvant treatment.

**Electronic supplementary material:**

The online version of this article (doi:10.1186/s12885-015-1793-9) contains supplementary material, which is available to authorized users.

## Background

Despite the incidence of gastric cancer has declined in the modern society for decades, it remains the fourth most common malignancy and the third leading cause of cancer related death worldwide with an estimated 951,600 new cases and 723,100 deaths occurring in 2012 [1]. A substantial proportion of gastric cancer patients are diagnosed at advanced stages, due to occult symptoms at early stages, whereas patients in Japan gain a 5-year overall survival as high as 76 %, attributing to the screening for early stage gastric cancer [2]. Currently, prognostic models for gastric cancer are mainly based on the TNM classification of International Union Against Cancer, composed of tumor depth, lymph node metastasis and distant metastasis. The outcomes for patients with similar pathological TNM stage can be very diverse because of the heterogeneity of this disease [3, 4]. Therefore, stratifying patients in the current TNM stage system by incorporation of the molecules involved in carcinogenesis of gastric cancer may lead to more accurate prediction of the clinical outcome.

Chemokines are a superfamily of small molecule proteins and selectively regulate the recruitment and activation of leukocyte subsets to preferential sites through chemotaxis [5]. CXCR2 is a member of the G-protein-coupled receptor superfamily and the receptor for chemokines with the presence or absence of ELR motif (Glu-Leu-Arg). The ELR positive CXC chemokines (such as CXCL1, CXCL2, CXCL3, CXCL5, CXCL6, CXCL8 and CXCL7) are potent promoters of angiogenesis [6, 7]. A number of studies have demonstrated that CXCR2 plays a pivotal role in tumor angiogenesis, proliferation and invasion [8–10]. In gastric cancer, CXCR2 was found to be associated with tumor progression and invasion [11, 12]. Thus, we hypothesized that the addition of CXCR2 to TNM staging system has the potential to provide more individualized risk stratification based on molecular characteristics of the tumor.

In this study, we investigated CXCR2 expression in patients with gastric cancer by immunohistochemistry and explored its associations with clinicopathological factors and prognosis. Moreover, we generated a predictive nomogram integrating CXCR2 expression, tumor depth, and lymph node metastasis to assess the risk score for 5-year overall survival (OS) of gastric cancer patients.

## Methods

### Patients

We retrospectively recruited 357 consecutive gastric cancer patients from Zhongshan Hospital, Fudan University. Gastrectomy plus standard D2 lymphadenectomy was performed by the same surgical team in 2008. None of these patients received any preoperative chemotherapy or radiotherapy. Baseline clinicopathological features of these patients including age, gender, tumor location, tumor size, tumor differentiation, Lauren classification, and TNM stage were collected. Tumor stage and differentiation grade were reassessed according to the 7th Edition of the UICC/AJCC TNM Staging System by two independent gastroenterological pathologists. Median age at surgery was 59 years (range 27–85), and 70 % of patients were male. Intestinal and diffuse histologic subtypes constituted 63 % and 37 % of cases, respectively. Lymph node metastasis was present in 64 % of patients. Patients were followed up until April 2014 with a median follow-up time of 41 months. Overall survival was defined as the interval between the date of surgery and the date of death or last visit. The study was approved by the Clinical Research Ethics Committee of Zhongshan Hospital, Fudan University and written informed consent was obtained from each patient.

### Tissue microarray, immunohistochemical staining and evaluation

Tissue microarray construction and immunohistochemistry protocol were described previously [13]. The primary antibody against human CXCR2 (Abcam, Cambridge, MA, USA; dilution 1:100) was applied in the procedure. The staining intensity and extent were scored independently by two gastroenterological pathologists (Z. Shen and H. Zhang) who were blind to the patients’ outcome using the semi-quantitative immunoreactivity scoring (IRS) system as described previously [14]. The immunohistochemical-stained sections were scanned at × 200 magnification and three independent microscopic fields with the strongest staining were captured by NIS-Element F3.2 software to guarantee representativeness and homogeneity. The staining intensity was graded as 0 (negative), 1 (weak), 2 (moderate), and 3 (strong) and the staining extent was scored as the percentage of positive cells (0–100 %). The staining intensity and extent were multiplied to obtain a CXCR2 immunohistochemical score on a scale of 0 to 300. The agreement among the two pathologists was excellent, which was evaluated by the kappa value (0.85). To dichotomize CXCR2 expression into high and low groups, the score of 200 was selected as the cutoff point according to the minimum *p*-value method based on its correlation with OS. The negative control staining was treated equally with the primary antibody excluded.

### Statistical analysis

SPSS 21.0 (SPSS Inc., IL, Chicago, USA) was used to perform the analyses. Correlations between immunohistochemical variables and clinicopathologic characteristics were analyzed with Pearson χ^2^ and Student’s *t* tests. Kaplan-Meier method with log-rank test was applied to compare survival curves. Cox regression models were used to analyze the impact of prognostic factors on OS. Nomogram was constructed by R software version 3.0.2 with “rms” package (R Foundation for Statistical Computing, Vienna, Austria). Calibration plot for 5-year overall survival was generated to assess the performance characteristics of the constructed nomogram. The Harrell’s concordance indices (c-indices) were calculated to evaluate the discrimination of different models for OS prediction. All statistical analyses were two-sided and *p* < 0.05 was regarded as statistically significant.

## Results

### CXCR2 expression and associations with clinicopathological features in gastric cancer patients

**Fig. 1:**
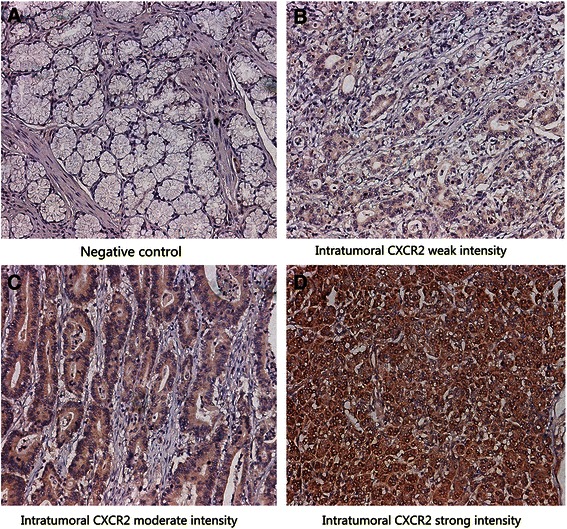
Expression of CXCR2 in sections of gastric cancer. Representative photographs of CXCR2 expression (**a**–**d**). Negative control **a**. Representative photographs of weak, moderate and strong staining (**b**–**d**). Original magnification: ×200

Immunohistochemical staining section analysis demonstrated that CXCR2 expression was mainly located in the cytoplasm of gastric carcinoma cells (Fig. 1b–d). The median intratumoral CXCR2 staining score was 210 (range 0–300). The negative control showed no staining neither in gastric epithelial cells nor in stroma cells (Fig. 1a). The relationships between clinical pathological characteristics and CXCR2 expression are shown in Table 1. High CXCR2 expression correlated with poor tumor differentiation (*p* = 0.021), increased tumor depth (*p* < 0.001), lymph node metastasis (*p* < 0.001) and advanced TNM stage (*p* < 0.001).

**Table 1:** Relation between intratumoral CXCR2 expression and clinical characteristics in patients with gastric cancer (*n* = 357)

Factor	Patients		CXCR2 expression		
	No.	%	Low	High	*P* value
All patients	357	100	157	200	
Age (years)					0.250
Mean ± SD^†^	59.4 ± 11.6		58.6 ± 11.1	60.1 ± 11.9	
Gender					0.477
Female	107	30	44	63	
Male	250	70	113	137	
Tumor size (cm)					0.931
Mean ± SD†	3.8 ± 2.1		3.8 ± 2.4	3.8 ± 1.9	
Differentiation					0.021
Well	20	5.6	14	6	
Moderately	127	35.6	60	67	
Poorly	210	58.8	83	127	
Lauren classification					0.910
Intestinal	224	62.7	98	126	
Diffuse	133	37.3	59	74	
pT stage					<0.001
T1	60	16.8	40	20	
T2	50	14.0	25	25	
T3	65	18.2	27	38	
T4	182	51.0	65	117	
pN stage					<0.001
N0	128	35.8	72	56	
N1	37	10.4	21	16	
N2	70	19.6	24	46	
N3	122	34.2	40	82	
Distant metastasis					0.735
Absent	349	97.8	153	196	
Present	8	2.2	4	4	
TNM stage					0.001
I	78	21.8	49	29	
II	80	22.5	37	43	
III	191	53.5	67	124	
IV	8	2.2	4	4	

*p < 0.05 was regarded as statistically significant. ^†^SD: standard deviation

### High expression of CXCR2 is associated with poor clinical outcome

**Fig. 2:**
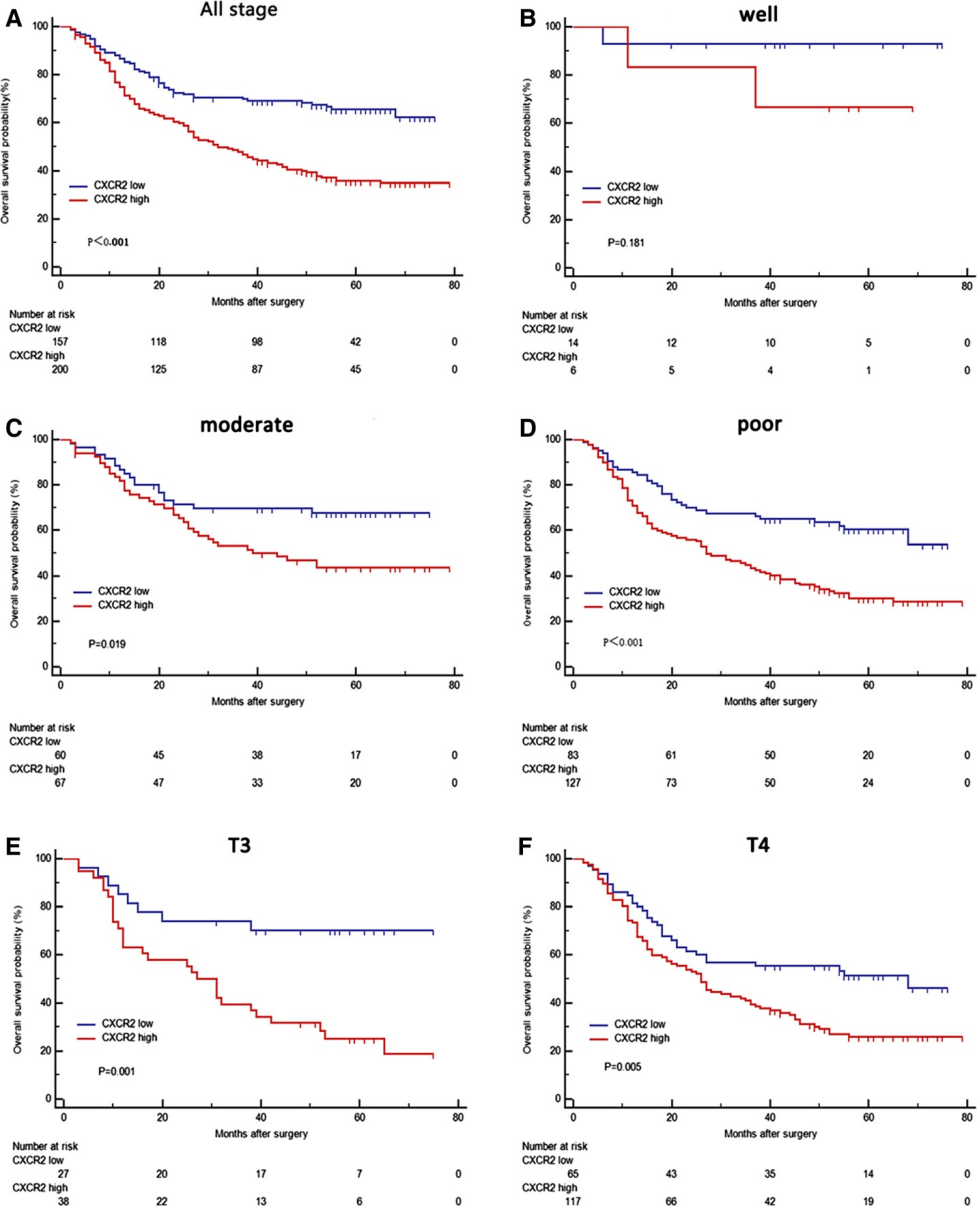
Kaplan–Meier analysis for OS of patients with gastric cancer according to the CXCR2 expression. **a** All patients, *n* = 357, *p* < 0.001. **b** Well differentiated tumors, *n* = 20, *p* = 0.181. **c** Moderately differentiated tumors, *n* = 127, *p* = 0.019. **d** Poorly differentiated tumors, *n* = 210, *p* < 0.001. **e** Tumors of T3 stage, *n* = 65, *p* = 0.001. **f** Tumors of T4 stage, *n* = 182, *p* = 0.005. *p* value was calculated by Log-rank test

The Kaplan-Meier curves revealed that high CXCR2 expression correlated with shorter OS (*p* < 0.001, Fig. 2a). The median survival time for CXCR2 high and low expression group was 32 and 51 months, respectively.

**Fig. 3:**
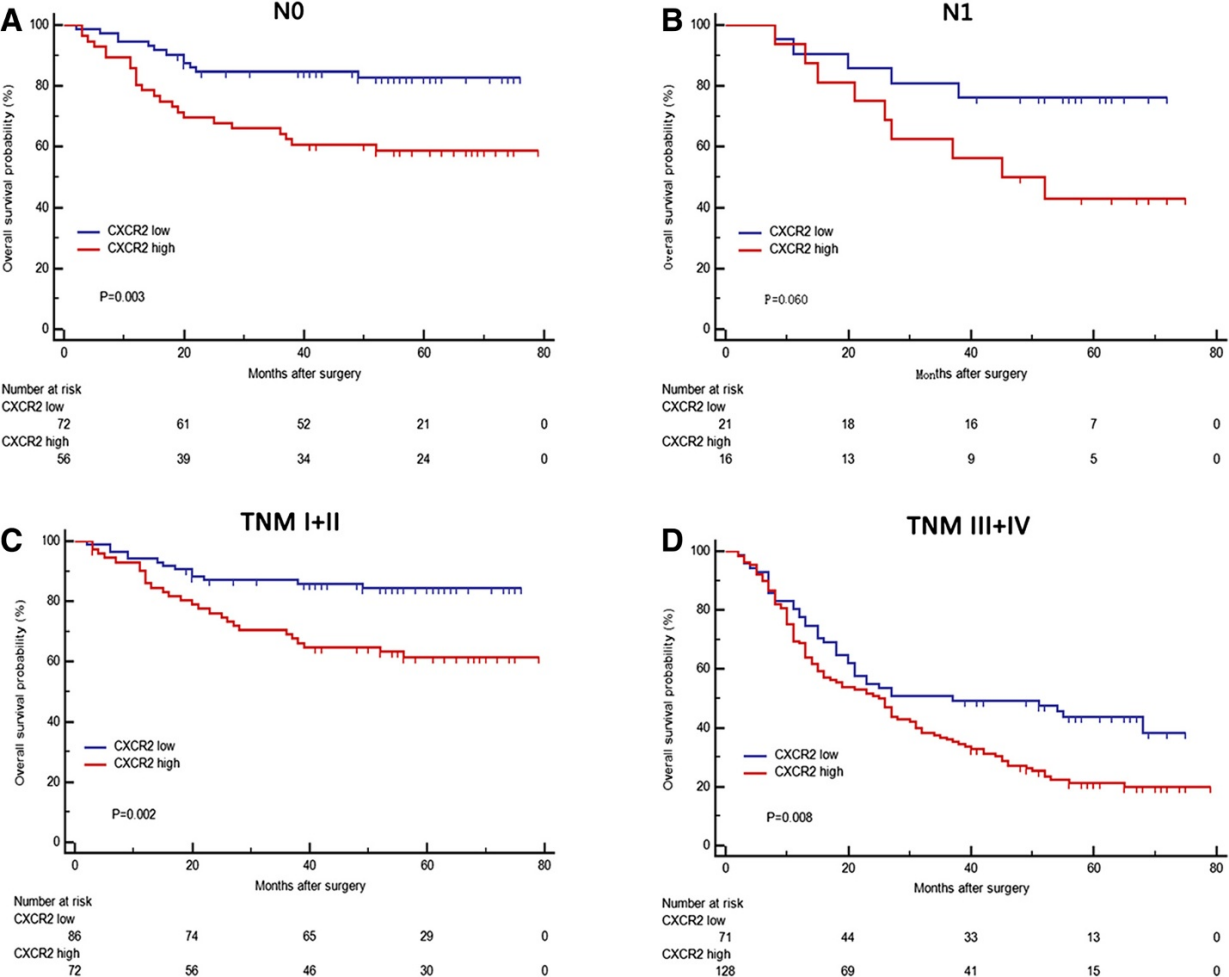
Kaplan–Meier analysis to assess prognostic value of CXCR2 in N0, N1 stage and different TNM stage. **a** Patients with N0 stage tumor, *n* = 128, *p* = 0.003. **b** Patients with N1 stage tumor, *n* = 37, *p* = 0.060. **c** Patients with TNM I + II stage tumor, *n* = 158, *p* = 0.002. **d** Patients with TNM III + IV stage tumor, *n* = 199, *p* = 0.008. *p* value was calculated by Log-rank test

Subgroup analysis revealed that intratumoral CXCR2 expression played an unfavorable prognostic role in patients of T3 (*p* = 0.001, Fig. 2e), T4 (*p* = 0.005, Fig. 2f), N0 (*p* = 0.003, Fig. 3a), moderate differentiation (*p* = 0.019, Fig. 2c), poor differentiation (*p* < 0.001, Fig. 2d), TNM I + II (*p* = 0.002, Fig. 3c), TNM III + IV (*p* = 0.008, Fig. 3d), Lauren intestinal type (*p* < 0.01, Additional file 1: Figure S1E) and Lauren diffuse type (*p* = 0.012, Additional file 1: Figure S1F). In contrast, intratumoral CXCR2 expression had limited ability to stratify patients with T1, T2, N1, N2, N3 and well differentiated disease. (Figure 2b, Additional file 1: Figure S1A-D). To further elucidate the predictive value of CXCR2 precisely, we calculated its hazard ratios (HR) using univariate COX regression in different subgroups and found that CXCR2 expression exerted the same adverse prognostic role as it did in Log-rank test (Additional file 2: Figure S2).

### Multivariate analysis and predictive nomogram for OS of gastric cancer patients

**Fig. 4:**
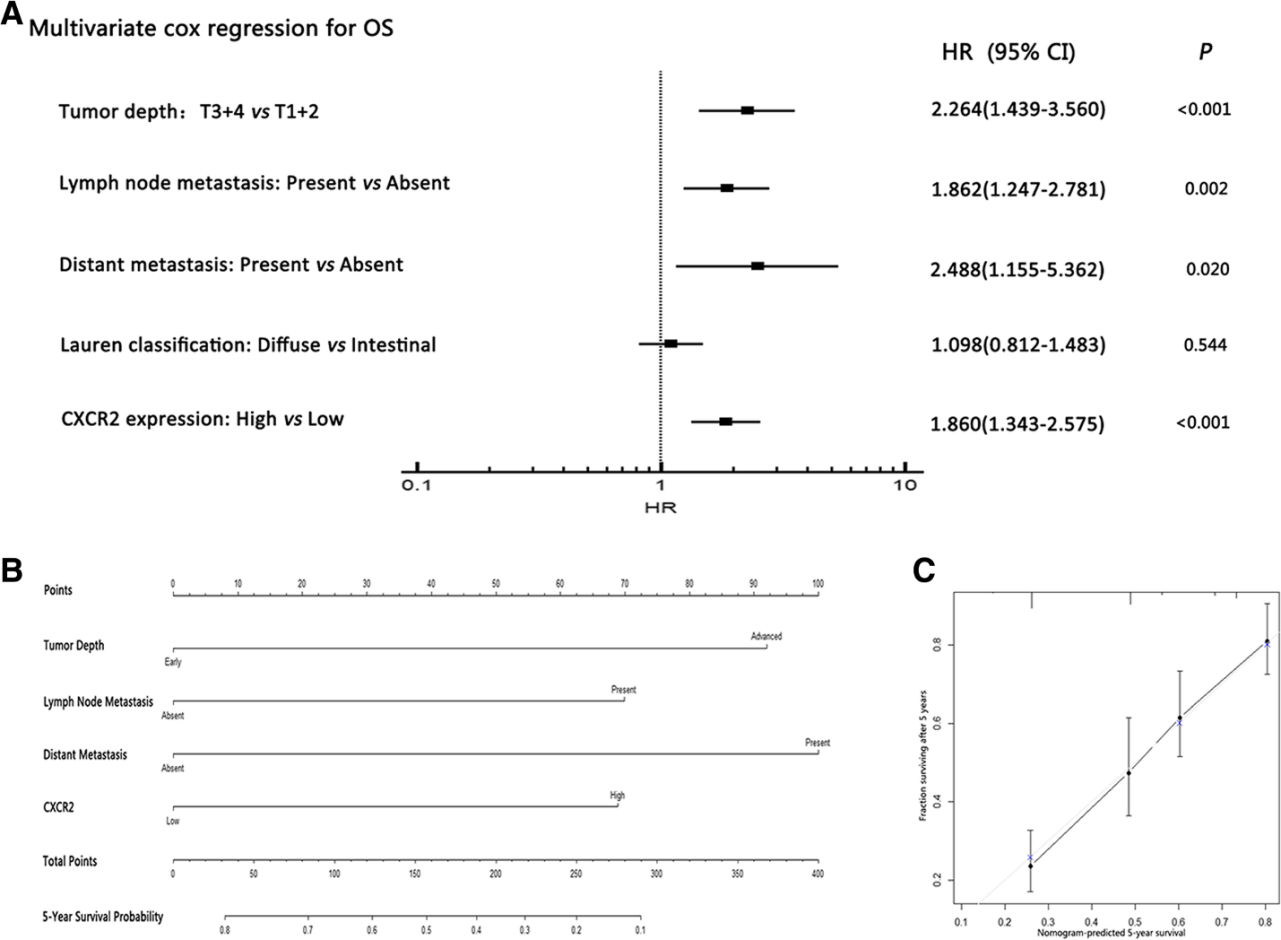
Multivariate analysis, nomogram and calibration plot for the predictive value of CXCR2 expression in patients of gastric cancer. **a** Multivariate Cox analysis identified independent prognosticators for OS of the cohort. **b** Nomogram constructed to predict 5-years overall survival in gastric cancer: Tumor invasion depth (early = T1 + T2, advanced = T3 + T4), Lymph node metastasis (absent = N0, present = N1 + N2 + N3), Distant metastasis (absent = M0, present = M1) and CXCR2 expression (low = low expression, high = high expression) were included. **c** Calibration plot for nomogram-predicted and observed 5-year survival. The nomogram performed well with the ideal model

We then evaluated the independent prognostic value of CXCR2 expression using multivariate Cox proportional hazard model. The results showed that the CXCR2 expression was independently prognostic of mortality (HR = 1.860; 95 % CI = 1.343-2.575; *p* < 0.001) in patients with gastric cancer after adjusting for established clinicopathologic factors (Fig. 4a).

Predictive nomogram was constructed using all the significant independent predictors for OS from Cox regression analysis. In the nomogram, the hazard ratio for each factor was turned into points, and a higher total points indicated worse survival overall probability (Fig. 4b). The calibration curve for predicted 5-year OS showed a good performance with the ideal model (Fig. 4c). The Harrell’s concordance index (c-index) for the nomogram constructed by TNM and CXCR2 expression was 0.664, higher than 0.642 of TNM alone.

## Discussion

CXCR2 expression has been implicated in gastric cancer progression [11, 12, 15, 16]. Coexpression of CXCL1 and CXCR2 acts like an autocrine or paracrine mechanism to actuate metastasis of gastric cancer [15]. However, its prognostic value in gastric cancer patients has not been well established. In this study, we investigated the expression of this chemokine receptor with immunohistochemistry on gastric cancer tissue microarray and its relationships with pathologic factors and prognosis. Our data showed a consistent result with previous studies that CXCR2 expression positively correlated with tumor depth, lymph node metastasis and TNM stage (Table 1), which implied that CXCR2 expression might synergize gastric cancer proliferation, invasion and metastasis.

Notably, an interesting phenomenon had been observed that CXCR2 staining increased gradually accompanied with gastric cancer differentiation from well to poor (Fig. 1b–d). This raised the possibility that CXCR2 expression increases during the dedifferentiation process of gastric cancer cells and might take on a particular role in gastric cancer differentiation. However, the biological mechanisms underlying this phenomenon merit further investigation.

The connection between inflammation and cancer was first noted by Virchwood in the 19th century [13]. Chronic inflammation is frequently present before several types of carcinogenesis. Chemokines are the key molecule for inducing leukocytes to inflammation or tumor site [17–19]. Expression of pro-inflammatory chemokines facilitates a chronic inflammation process and helps establish a favorable tumor milieu, which stimulates tumor proliferation and invasion *via* their receptors on tumor cells [20, 21]. Many studies have identified that ELR positive chemokines play a pleiotropic role in inflammation, angiogenesis, carcinogenesis and metastasis [22–24].

CXCR2 was reported to play a critical role in a range of cancers, such as colon cancer [25], oral squamous cell cancer [26], esophageal cancer [27] and breast cancer [28]. CXCR2 had been found to be the primary functional chemokine receptor in mediating endothelial cell chemotaxis [29]. All ELR+ CXC chemokine ligands, binding to CXCR2, mediated angiogenic activity, which was crucial for cancer cells proliferation [22]. Heidemann found that after activation of CXCR2 using interleukin-8 (IL-8), endothelial cells gained enhanced capacity of fiber assembly, proliferation and phosphorylation of its downstream signaling molecule ERK1/2 while this phenomenon could be impaired by either using specific antibodies to CXCR2 or inhibitor for ERK1/2 [24, 25]. The importance of CXCR2 in angiogenesis *in vivo* had also been proven in the cornea micropocket assay by CXCR2 knockout mice [22]. Thus, the correlation between aberrant expression of CXCR2 and the poor prognosis of the patients was possibly due to its angiogenic role in gastric cancer.

Although several biomarkers have been introduced to the prognosis models for gastric cancer recently [30,31], conventional predictive models majorly rely on TNM stage, which has limited ability to discriminate a stratum of patients for the heterogeneity of this disease. Kaplan-Meier and univariate COX stratification analysis revealed that CXCR2 had a discriminatory power in most subgroups of different clinicopathological types (Additional file 2: Figure S2). Further analysis of multivariate COX regression verified that CXCR2 bears an independent prognostic value, which could be integrated to the TNM staging system in the nomogram (Fig. 4a–c). Validation test using calibration plot and c-index indicated that this nomogram performed better than the TNM stage alone.

Giving the prognostic value of CXCR2 expression in gastric cancer, optimal use of CXCR2 inhibitors would be a potential choice of adjuvant therapy for gastric cancer patients after gastrectomy. However, due to the retrospective design in nature and the relatively small size of the patient population, a multicenter, prospective study is needed to validate these results in a larger population in the future.

## Conclusion

Our present study identified that intratumoral CXCR2 expression correlates with gastric cancer progression, tumor differentiation and lymph node metastasis and can be utilized as a novel prognostic factor for patient outcomes. Incorporating CXCR2 expression into TNM stage can provide a better prognostic model for patients with gastric cancer. Inhibition of CXCR2 might be a promising target of postoperative adjuvant therapy modality for gastric cancer patients.

## Additional files

Additional file 1: Figure S1. Kaplan–Meier analysis to assess prognostic value of CXCR2 in some clinicopathological factors. (A) T1 stage, *n* = 80, *p* = 0.386. (B) T2 stage, *n* = 50, *p* = 0.803. (C) N2 stage, *n* = 70, *p* = 0.124. (D) N3 stage, *n* = 122, *p* = 0.162. (E) Lauren intestinal type, *n* = 224, *p* < 0.01. (F) Lauren diffuse type, *n* = 133, *p* = 0.012. (JPEG 322 kb)
Additional file 2: Figure S2. COX analysis assesses prognostic value of CXCR2 with hazard ratios for OS in subgroups. T3 (*n* = 65, HR: 3.326, 95 % CI: 1.522-7.267, *p* = 0.003), T4 (*n* = 182, HR: 1.768, 95 % CI: 1.178-2.652, *p* = 0.006), N0 (*n* = 128, HR: 2.782, 95 % CI: 1.389-5.574, *p* = 0.004), TNM I + II (*n* = 158, HR: 2.713, 95 % CI: 1.404-5.241, *p* = 0.003), TNM III + IV (*n* = 199,HR: 1.623, 95 % CI: 1.126-2.340, *p* = 0.01), well and moderate differentiation (*n* = 147, HR: 2.159, 95 % CI: 1.262-3.691, *p* = 0.005), poor differentiation (*n* = 210, HR: 2.158, 95 % CI: 1.448-3.217, *p* < 0.001), Lauren intestinal type (*n* = 224, HR: 2.573, 95 % CI: 1.672-3.958, *p* < 0.001), Lauren diffuse type (*n* = 133, HR: 1.834, 95 % CI: 1.137-2.960, *p* = 0.014). (JPEG 302 kb)

## Abbreviations

CXCR2: C-X-C chemokine receptor 2
CXCL: C-X-C chemokine ligand
OS: Overall survival
UICC: International Union Against Cancer
AJCC: American Joint Committee on Cancer
IL8: Interleukin 8
ERK1/2: Extracellular signal-regulated kinase1/2
c-index: Harrell’s concordance index
