# Supplementary figures and images for: The prognostic value of CXC-chemokine receptor 2 (CXCR2) in gastric cancer patients
Source: BMC Cancer. 2015 Oct 23;15:766. doi: 10.1186/s12885-015-1793-9 (PMC4619066; doi:10.1186/s12885-015-1793-9)

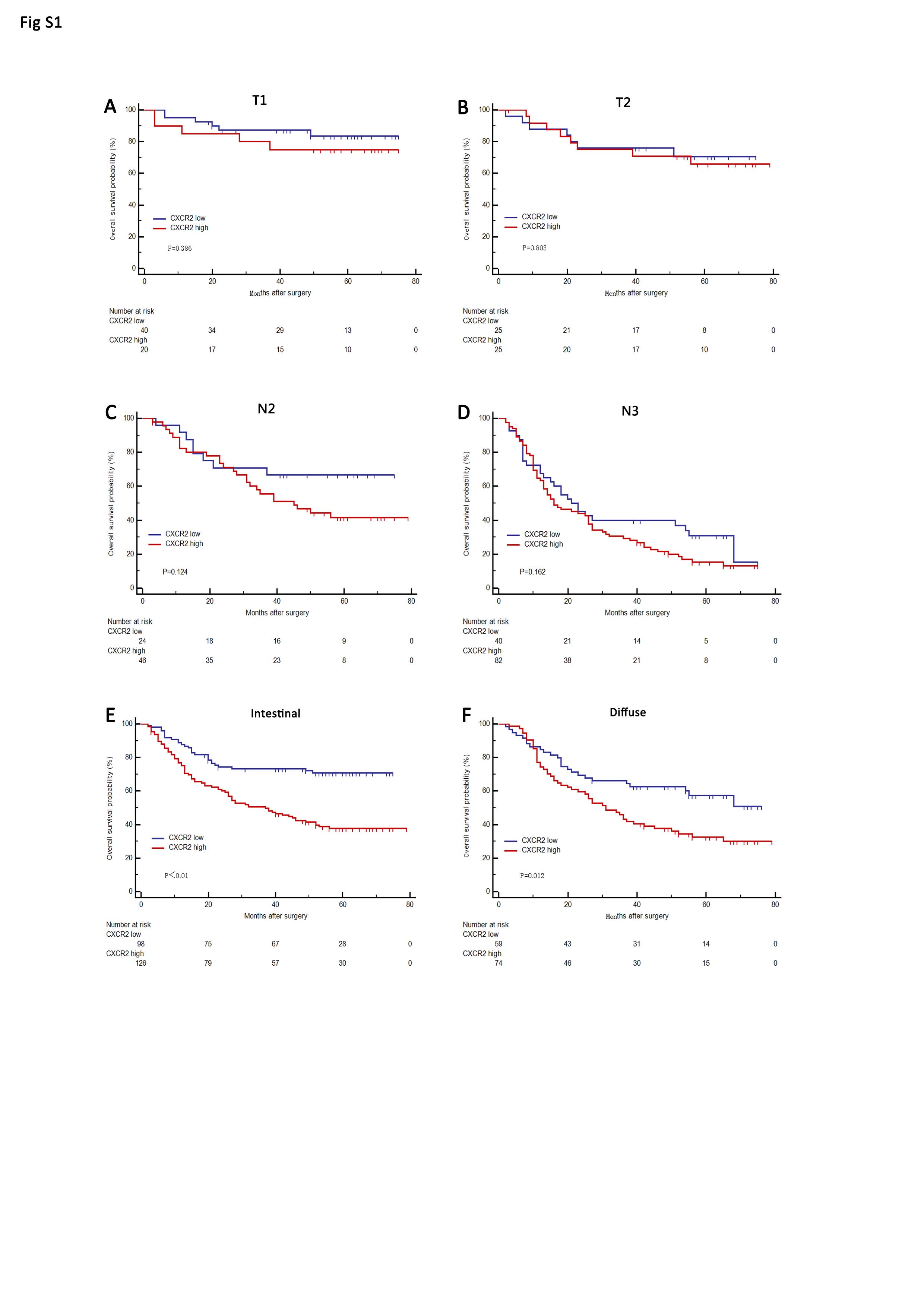

Supplement: Additional file 1: Figure S1. — Kaplan–Meier analysis to assess prognostic value of CXCR2 in some clinicopathological factors. (A) T1 stage, n = 80, p = 0.386. (B) T2 stage, n = 50, p = 0.803. (C) N2 stage, n = 70, p = 0.124. (D) N3 stage, n = 122, p = 0.162. (E) Lauren intestinal type, n = 224, p < 0.01. (F) Lauren diffuse type, n = 133, p = 0.012. (JPEG 322 kb) [file 12885_2015_1793_MOESM1_ESM.jpeg]

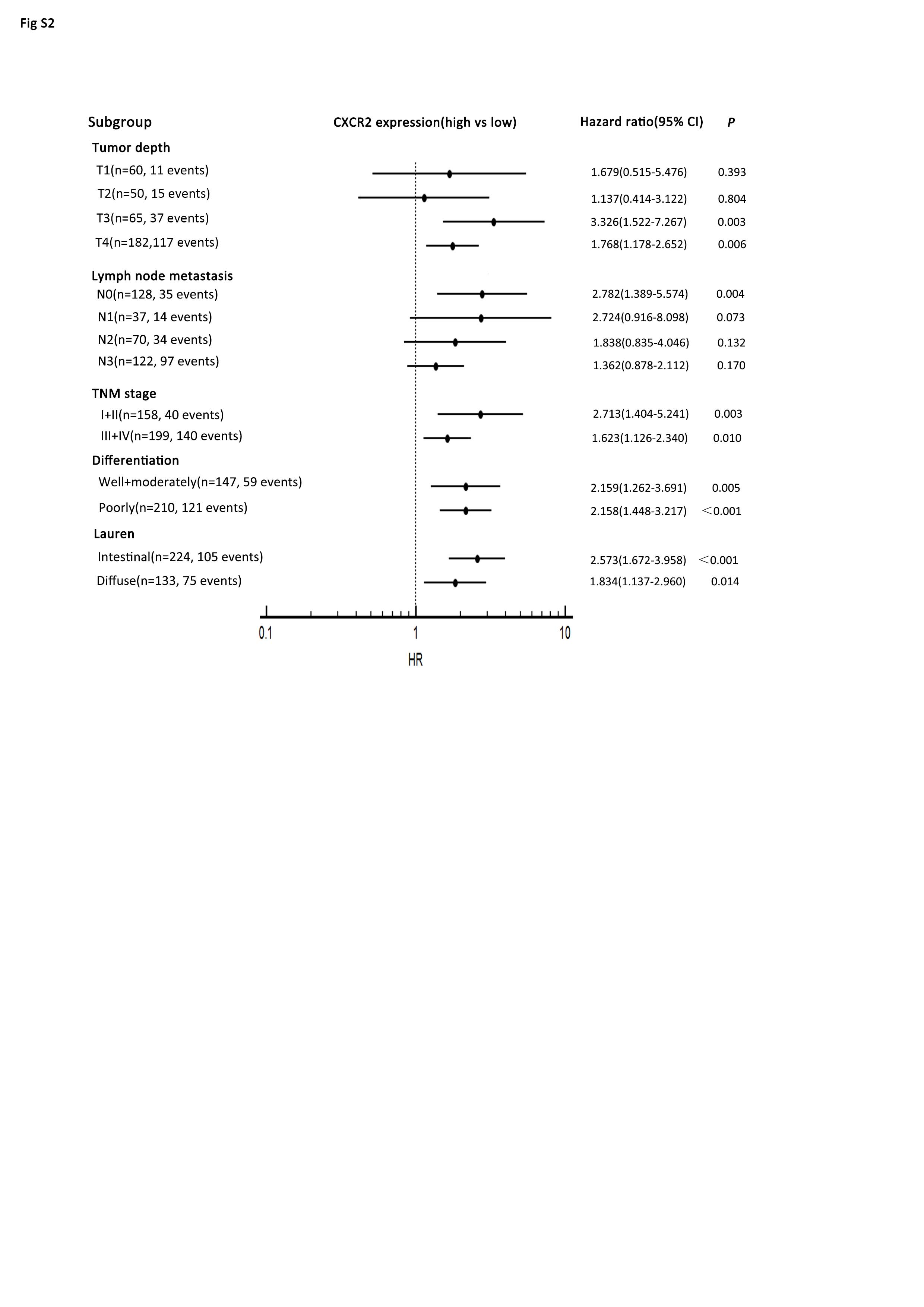

Supplement: Additional file 2: Figure S2. — COX analysis assesses prognostic value of CXCR2 with hazard ratios for OS in subgroups. T3 (n = 65, HR: 3.326, 95 % CI: 1.522-7.267, p = 0.003), T4 (n = 182, HR: 1.768, 95 % CI: 1.178-2.652, p = 0.006), N0 (n = 128, HR: 2.782, 95 % CI: 1.389-5.574, p = 0.004), TNM I + II (n = 158, HR: 2.713, 95 % CI: 1.404-5.241, p = 0.003), TNM III + IV (n = 199,HR: 1.623, 95 % CI: 1.126-2.340, p = 0.01), well and moderate differentiation (n = 147, HR: 2.159, 95 % CI: 1.262-3.691, p = 0.005), poor differentiation (n = 210, HR: 2.158, 95 % CI: 1.448-3.217, p < 0.001), Lauren intestinal type (n = 224, HR: 2.573, 95 % CI: 1.672-3.958, p < 0.001), Lauren diffuse type (n = 133, HR: 1.834, 95 % CI: 1.137-2.960, p = 0.014). (JPEG 302 kb) [file 12885_2015_1793_MOESM2_ESM.jpeg]
